# Analysis of drug pricing drivers under South Korea’s pharmaco-economic evaluation exemption policy (2015–2022)

**DOI:** 10.3389/fphar.2024.1519491

**Published:** 2025-01-07

**Authors:** Seung Rae Yu, Jong Hyuk Lee

**Affiliations:** ^1^ College of Pharmacy, Dong-Duk Women’s University, Seoul, Republic of Korea; ^2^ College of Pharmacy, Chung-Ang University, Seoul, Republic of Korea

**Keywords:** pricing, health technology assessment, drug price, budget impact, cost-effectiveness, patient accessibility

## Abstract

**Objective:**

This study analysed the characteristics of new drugs listed under the pharmaco-economic evaluation exemption (PEE) system from 2015 to 2022 in South Korea and examined the factors influencing the pricing decisions under this system.

**Methods:**

A mixed-methods statistical approach was used to comprehensively evaluate the factors influencing drug pricing under PEE system. Descriptive statistics provide an overview of the dataset, while inferential statistics, including t-tests and Pearson’s correlation analyses, are used to explore variable associations. Multiple and hierarchical regression models identify and quantify the key determinants of drug prices, controlling for multicollinearity among the variables.

**Results:**

From 2015 to 2022, 30 new drugs were listed under the PEE system. The average annual number of new drugs was four, but this figure significantly increased to eight in 2022. The “KOR/A7 lowest” variable exhibited a strong negative correlation with the budget impact variable (coefficient: 0.838, *P* < 0.001), indicating that drugs with higher budget impact tend to have lower prices compared to the A7 countrie’s lowest price.

**Conclusion:**

Since the introduction of the PEE system in South Korea, patient access to new drugs has significantly improved. However, the rising expenditure on pharmaceuticals has made budget impact a significant consideration in pricing decisions, highlighting the need for ongoing monitoring of drug expenditure by payers. As the system evolves, enhanced oversight and policy adjustments will be crucial for balancing cost containment with equitable patient access. Developing tiered RSA models based on drug classification or therapeutic impact could be a viable approach to achieving this balance.

## 1 Introduction

The development of innovative medicines for serious diseases, such as cancer and rare diseases, is advancing globally, thereby enhancing the potential pool of treatment for patients. These medicines target diseases that are difficult to treat with existing therapies and offer new options that contribute to improved survival rates and quality of life. However, these innovative drugs are often extremely expensive, leading to a sharp increase in pharmaceutical spending for both patients and payers. It also raises concerns and debate over the efficient allocation of resources (Pan et al., 2024; Risse et al., 2024; Stewart et al., 2024). These new drugs frequently target small patient populations for which no alternative treatments are available, making it challenging to demonstrate their clinical usefulness. Consequently, generating sufficient evidence to prove cost-effectiveness is challenging (Buckley, 2008; van Overbeeke et al., 2021). Specifically, the high cost of treatment for rare diseases or cancer often results in significant uncertainty regarding their clinical utility relative to their expense, making it difficult to demonstrate cost-effectiveness through economic evaluations (Clifford et al., 2024). As a result, the reimbursement process for newly approved drugs that have undergone lengthy developmental and regulatory approval is often delayed, leading to situations where patients cannot access these new treatments. This situation may result in patients bearing the high cost of these medications, ultimately reducing their access to new treatments.

Additionally, if new drug sales underperform, pharmaceutical companies may not receive sufficient returns on their research and development (R&D) investments, potentially negatively impacting the promotion of new drug technology development (Chen and Shao, 2023). Therefore, many countries have implemented various measures to enhance access to new innovative high-cost drugs. Various international programmes, such as the UK’s EAMS and France’s ATU, aim to expedite patient access to critical therapies for severe rare diseases with no alternatives (government, U. Guidance, 2024; Jacquet et al., 2023). Flexibility in ICER thresholds, as seen in the UK’s Highly Specialized Technologies programme, further supports the reimbursement of high-cost medicines (NICE NICE health technology evaluations, 2024). Alternative evaluation methods, such as multicriteria decision analysis (MCDA), have been proposed to address the limitations of traditional economic evaluations (Lasalvia et al., 2019). MCDA considers multiple factors beyond cost-effectiveness, providing a more comprehensive approach to evaluating the value of new treatments.

Given the absence of alternative treatments and the high clinical necessity of these medications, the South Korean government faces challenges in negotiating prices with multinational pharmaceutical companies. This highlights the necessity of exploring flexible pricing strategies under the PEE system to address financial sustainability while ensuring patient access.

Additionally, separate funds have been established to enhance access to new treatments, such as Australia’s Life Saving Drugs Program (LSDP), Canada’s New Drug Funding Program (NDFP), the UK’s New Cancer Drugs Fund (New CDF), and Italy’s 5% AIFA Fund (Berry et al., 2007; Morrell et al., 2018; Taylor et al., 2018; Xoxi et al., 2021). These funds are managed outside of general public finances and are specifically dedicated to improving access to innovative medicines.

South Korea is also working to enhance access to high-cost innovative medicines through various measures, including essential medicines for clinical use, risk-sharing agreements (RSA), pharmacoeconomic evaluation exemptions (PEE), and increasing the ICER threshold (Yoo et al., 2019; Kim et al., 2021a). In particular, the PEE system, introduced in 2015, allows for the omission of cost-effectiveness proofs for rare disease treatments or cancer drugs that lack alternatives and have a small patient population, provided that the drug is listed in at least three of the A7 countries (US, UK, Germany, France, Italy, Switzerland, and Japan) (Yoo et al., 2019). The ‘KOR/A7 lowest’ ratio compares drug prices in Korea to the lowest price among the A7 countries, serving as a benchmark for price negotiations under the PEE system.

In South Korea, innovative new drugs, including one-shot therapies, are reimbursed through the PEE system. However, both pharmaceutical companies and patients argue for expanding the scope of PEE to enhance access to new treatments (Kim et al., 2023). Consequently, there is a need to study the specific status and characteristics of new drugs reimbursed under the PEE system in South Korea, as existing research has not specifically analysed the pricing drivers under this system. Previous studies have focused on new drug listing rates and timelines, such as the time required for listing in 2015, without examining PEE-reimbursed drugs separately (Kim et al., 2021b). Another study analyzed rare drug listing rates and timelines from 2012 to 2021, highlighting various systems for rare drug access, including pharmacoeconomic evaluations, weighted average price comparisons, risk-sharing agreements, and PEE systems (Hwang et al., 2023). However, Hwang et al. primarily focused on patient out-of-pocket costs, leaving a gap in understanding pricing factors and the broader impact of the PEE system.

Our study examines the characteristics of new drugs listed under the PEE system in South Korea from 2015 to 2022 and investigates the key factors influencing pricing decisions. Additionally, we aim to provide actionable insights for improving accessibility to innovative medicines.

## 2 Materials and methods

### 2.1 Data collection

Data were extracted from publicly accessible government databases in South Korea for the study period from January 2015 to December 2022 (Service, 2024a; Service, 2024b). The Health Insurance Reimbursement Drug List, Drug Reimbursement Evaluation Committee Results Disclosure, and Drug Price Negotiation Results were obtained from the websites of the Ministry of Health and Welfare (MOHW), the Health Insurance Review and Assessment Service (HIRA), and the National Health Insurance Service (NHIS) (Welfare, 2024a; Welfare, 2024b; Service, 2024c; Service, 2024d). The EZDRUG database of the Korean Ministry of Food and Drug Safety (KMFDS) was used to verify whether the drugs were designated as orphans or had oncological indications (Safety, 2024). Information related to pharmaceutical companies was obtained from the websites of the Korea Research-based Pharma Industry Association (KRPIA) and the Korea Pharmaceutical and Bio-Pharma Manufacturers Association (KPBMA).

The prices of new drugs listed under the PEE system in South Korea, as well as the number of A7 and OECD countries where these drugs are listed, were verified using websites specified by South Korean regulations (Association, 2024a; Association, 2024b). The estimated budget impact values were referenced from publicly disclosed results of the pricing negotiations between pharmaceutical companies and the NHIS, as listed in the Health Insurance Reimbursement Drug List by the MOHW.

#### 2.1.1 Independent variables

Factors mainly considered when determining the price of new drugs were selected as the following independent variables.

Drugs for severe diseases: indicate drugs are used for rare diseases or cancer.

Alternatives: indicates whether the drugs with an equivalent therapeutic level are available.

Number of patients: indicate the number of patients with the target disease.

Number of A7 countries listed: indicates the number of countries listed among the advanced countries (the USA, the UK, Germany, France, Italy, Switzerland, and Japan).

Budget impact: indicates the expected annual claim amount against the new drugs.

Listing period: indicates the period between the marketing authorization date and the listing date.

The ERP ratio was calculated as the ratio of the unit price in Korea to the lowest unit price among A7 countries, while the Price Level was defined as the ratio of the Korean price to the average A7 price.

### 2.2 Analysis method and variables

This study employs a mixed-methods statistical approach to comprehensively evaluate the factors influencing drug pricing. Descriptive statistics provide an overview of the dataset, while inferential statistics, including t-tests and Pearson’s correlation analyses, are used to explore variable associations. Multiple and hierarchical regression models identify and quantify the key determinants of drug prices, controlling multicollinearity among variables.

The variables used in the analysis were as follows:

(1) Origin of licensing holder: domestic or multinational company
(2) Indication: orphan or non-orphan drug; cancer, non-cancer, or orphan and cancer drug
(3) RSA: none, expenditure cap type, or expenditure cap type and refund type
(4) Budget: budget level (classified based on an estimated annual budget of 10,000 mil. KRW) or budget impact
(5) Price: price level (classified based on the average price ratio [65%] of Korean drug prices to foreign country prices).
(6) External reference price (ERP):
  • ERP list: Number of listed A7 and OECD countries
(7) ERP Ratio: Ratio of Korean drug prices to those of A7 and OECD countries
  • KOR/A7 lowest: Ratio of Korean drug prices to the lowest A7 countries price
  • KOR/A7: Ratio of Korean drug prices to the average A7 countries price
  • KOR/OECD: Ratio of Korean drug prices to the average OECD countries price
  • KOR/Total: Ratio of Korean drug prices to the overall average of A7 and OECD countries price
(8) Time period
  • 2015–2018: The period before the announcement of the health insurance coverage expansion policy.
  • 2019–2022: The period after the announcement of the health insurance coverage expansion policy in 2019.

### 2.3 Statistical analysis

The statistical models applied aimed to identify key drivers influencing drug pricing under the PEE system. Hierarchical regression analysis was employed to control multicollinearity and to assess the incremental contribution of each independent variable to the prediction of drug pricing. Data was analyzed using IBM SPSS Statistics for Windows, version 27.0. The significance level was set at *P* < 0.05.

## 3 Results

### 3.1 Analysis of PEE new drug listing status and characteristics by year

From 2015 to 2022, 30 new drugs were listed under the PEE system. The average annual number of new drugs was four, but this figure significantly increased to eight in 2022. Table 1 presents the status of new drugs before and after 2019 based on annual data. Significant increases were observed in the use of Hybrid RSA Contracts (Expenditure CAP + Refund type) and the listing of high-cost drugs (exceeding 10 billion KRW) after 2019. The Mann-Whitney *U* test revealed that the increase in budget per drug after 2019 was statistically significant (*P* = 0.008), with the average budget per drug increasing from 5,480 million KRW to 16,406 million KRW.

**TABLE 1:** New PEE drug listing status and characteristics by year.

Variables		2015 (n = 2)	2016 (n = 5)	2017 (n = 5)	2018 (n = 4)	2019 (n = 3)	2020 (n = 2)	2021 (n = 1)	2022 (n = 8)	Total (n = 30)	*P*
Company	Domestic		1	1		1	1			4	0.648
		2				2					
	Multinational	2	4	4	4	2	1	1	8	26	
		14				12					
Indication	Non-Orphan							1	1	2	0.442
		0				2					
	Orphan& Non-Cancer		2	1	2	1	1		2	9	
		5				4					
	Orphan& Cancer	2	3	4	2	2	1		5	19	
		11				8					
RSA	None	1	3							4	<0.001
		4				0					
	Expenditure Cap	1	2	5	4	1	1		1	15	
		12				3					
	Expenditure Cap& Refund					2	1	1	7	11	
		0				11					
Budget level^a^	low	2	4	5	4	1	2		4	22	0.012
		15				7					
	High		1			2		1	4	8	
		1				7					
Price level^b^	low	1		4	3	1	1		3	13	0.484
		8				5					
	High	1	5	1	1	2	1	1	5	17	
		8				9					
Budget Impact^c^	Sum	9,672	39,212	23,418	15,370	60,337	11,033	22,740	135,578	317,362	0.008
		87,672				229,688					
	Mean	4,837	7,842	4,684	3,843	20,112	5,517	22,740	16,947	10,579	
		5,480				16,406					
	SD	1,839	3,788	2,385	2,655	14,492	5,115	-	21,982	13,376	
		3,134				17,866					
	Mean Rank	11.50				20.07					
	Ranks Sum	184.0				281.0					
ERP List^d^	A7	6.5	4.8	5.2	4.5	5.0	5.5	7.0	5.0	5.1	0.735
		5.1				5.2					
	OECD^6^	10.0	8.4	11.2	10.3	11.0	12.5	19.0	9.9	10.5	0.487
		9.9				11.1					
	Total	16.5	13.2	16.4	14.8	16.0	18.0	26.0	14.9	15.6	0.512
		15.0				16.4					
ERP Ratio^e^	KOR/A7 (lowest)	98.0%	95.6%	95.4%	95.0%	96.2%	99.5%	94.3%	96.0%	96.0%	0.557
		95.7%				96.4%					
	KOR/A7	63.6%	70.5%	59.0%	50.1%	72.3%	69.6%	71.5%	64.5%	64.0%	0.168
		61.0%				67.4%					
	KOR/OECD	63.7%	75.8%	62.8%	52.1%	67.2%	74.6%	63.2%	66.1%	65.7%	0.527
		64.3%				67.3%					
	KOR/Total	63.6%	74.1%	61.1%	51.5%	68.9%	72.1%	65.3%	65.6%	65.0%	0.365
		63.1%				67.2%					

RSA, risk-sharing agreements; ERP, external reference price; KOR, Korea; A7, advanced countries (United States, United Kingdom, Italy, Germany, Japan, Switzerland, France).

Note. Chi-square tests were conducted for “Company,” “Indication,” “RSA,” “Budget Level,” “Price Level,” and “ERP, List.” Mann-Whitney *U* test was used for ‘Budget Impact,’ and t-tests for “ERP, Ratio.”

^a^ Budget Level: classified based on the estimated annual budget of 10,000 mil. KRW (1 mil. KRW ≈ USD 1,000).

^b^ Price level: classified based on the average price ratio (65%) of Korean drug prices to foreign countries.

^c^ Budget Impact: values are derived from annual estimated sales data submitted during NHIS pricing negotiations, as disclosed by MOHW, and are presented in million KRW, to maintain consistency with South Korean pricing regulations.

^d^ ERP List: number of listed countries, classified as A7 and OECD countries.

^e^ ERP Rat[io: KOR/A7 (lowest) compares Korean drug prices to the lowest A7 country price, KOR/A7 to the average A7 price, KOR/OECD to the average OECD price, and KOR/Total to the overall average of A7 and OECD, prices.

Analysis of the expected budget agreed upon by the NHIS and pharmaceutical companies for new PEE drugs yielded the following results: The total sum of the annual estimated budget for new drugs listed under the PEE system from 2015 to 2022 amounted to 317,362 million KRW, with an average of 10,579 million KRW per drug. The budget per drug increased approximately threefold from before to after 2019, and this increase was statistically significant (Mann-Whitney *U* test, *P* = 0.008).

Analysis of the ERP variables confirmed that new PEE drugs listed in South Korea were, on average, listed in 15 countries, including five A7 countries and 10 other countries. The Korean price accounted for 65.0% of the overall foreign price, with 64.0% compared to the A7 countries and 65.7% compared to other countries. The Korean price relative to the A7 minimum price averaged 96.0%, suggesting successful price reductions through NHIS negotiations. While the ratio exhibited an upward trend post-2019, the change was not statistically significant (*P* = 0.557). Despite the lack of statistical significance, it is noteworthy that the ratio of Korean prices to foreign prices increased across all indicators after 2019.

### 3.2 Correlation analysis

Table 2 presents the results of the correlation analysis for the continuous variables. The ERP List variable showed no significant correlation with the ERP ratio or the budget impact. However, the “KOR/A7 lowest” variable exhibited a strong negative correlation with the budget impact (r = −0.492, *P* < 0.001). Conversely, the “KOR/A7” variable showed a positive correlation with the budget impact variable. These findings indicate that drugs with higher budget impact are priced closer to the A7 average price, suggesting that financial considerations significantly influence pricing decisions. Additionally, drugs with a significant budget impact tended to have prices closer to the A7 average price than other drugs.

**TABLE 2:** Correlation analysis between ERP and budget variables.

Variables		Mean	SD	ERP list			ERP ratio				Budget impact
				A7	Rest	Total	KOR/A7 (lowest)	KOR/A7	KOR/Rest	KOR/Total	
ERP List^a^	A7	5.13	1.20	1							
	Rest	10.50	4.64	0.690^**^	1						
	Total	15.63	5.53	0.795^**^	0.988^**^	1					
ERP Ratio^b^	KOR/A7 (lowest)	96.0%	3.2%	0.127	0.033	0.055	1				
	KOR/A7	64.0%	12.7%	0.022	−0.091	−0.072	−0.157	1			
	KOR/OECD	65.7%	12.7%	−0.192	−0.131	−0.151	−0.048	0.792^**^	1		
	KOR/Total	65.0%	12.2%	−0.116	−0.119	−0.125	−0.104	0.914^**^	0.969^**^	1	
Budget Impact^c^		10,579	13,376	−0.183	−0.056	−0.086	−0.492^**^	0.372^*^	0.308	0.346	1

*P < 0.05, **P < 0.005, ***P < 0.001.

ERP, external reference price; KOR, Korea; A7, advanced countries (United States, United Kingdom, Italy, Germany, Japan, Switzerland, France).

^a^ ERP List; The number of countries where the drug is listed, classified as A7 and OECD countries.

^b^ ERP Ratio: KOR/A7 (lowest) compares Korean drug prices to the lowest A7 country price; KOR/A7 to the average A7 price; KOR/OECD to the average OECD price; and KOR/Total to the overall average of A7 and OECD prices.

^c^ Budget Impact: Mean and SD values (in million KRW) represent the estimated annual sales reported during pricing negotiations between pharmaceutical companies and the NHIS, as disclosed by MOHW.

### 3.3 Multiple regression analysis for the “KOR/A7 lowest” price ratio

A multiple regression analysis was conducted to identify the variables that influence the pricing decisions for new PEE drugs (Table 3). The dependent variable was set as “Korean Price compared to A7 Lowest Price”. The multiple regression model identified significant predictors of the “KOR/A7 Lowest” price ratio. The budget impact variable had the highest standardized regression coefficient (−0.838, *P* < 0.001), indicating that higher financial impact corresponds to lower pricing relative to the A7 minimum price. As a result, the variance inflation factor (VIF) values for ‘KOR/A7’, “KOR/rest”, and “KOR/ERP” were found to be 48, 130, and 294, respectively, indicating high multicollinearity. Therefore, these variables were excluded as independent variables. For the ERP List variable, only the A7 and rest variables were included to avoid duplication.

**TABLE 3:** Results of multiple regression analysis.

Variables		Full model					Stepwise model					
		B	*β*	t(p)^d^	TOL^c^	VIF^c^	B	*β*	t(p)^d^	TOL^c^	VIF^c^	
Company	Domestic	Reference										
	Multi national	−0.018	−0.200	−1.083	0.608	1.645						
Period	Before 2019	References										
	After 2019	0.003	0.046	0.164	0.261	3.836						
ERP List^a^	A7	−0.001	−0.026	−0.121	0.439	2.279						
	OECD	0.000	0.048	0.234	0.488	2.049						
Indication	Non-Orphan	References										
	Orphan and Non-Cancer	0.007	0.104	0.302	0.173	5.778						
	Orphan and Cancer	0.014	0.214	0.695	0.218	4.587						
RSA	None	References					References					
	Expenditure Cap	−0.022	−0.345	−1.411	0.346	2.887						
	Expenditure Cap and Refund	0.020	0.312	0.827	0.145	6.887	0.039	0.595	3.769^**^	0.737	1.356	
Budget Impact^b^		−2.000 × 10^−6^	−0.838	−4.634^***^	0.631	1.584	−1.902 × 10^−6^	−0.797	−5.048^***^	0.737	1.356	
Constant		0.988		26.664^***^			0.966		168.646^***^			
F statistic		3.158^*^					13.690^***^					
Adjusted *R*-squared		0.401					0.467					
Durbin-Watson		2.54					2.384					

*P < 0.05, **P < 0.005, ***P < 0.001.

B, unstandardized regression coefficients; β, standardized regression coefficients; RSA, risk-sharing agreements; ERP, external reference price; A7, advanced countries (United States, United Kingdom, Italy, Germany, Japan, Switzerland, France).

^a^ ERP List: The number of listed countries, classified as A7 and OECD, countries.

^b^ Budget Impact: Values are described as million KRW (Sum, Mean, Rank statistics). Budget Impact represents the estimated annual sales reported during pricing negotiations between pharmaceutical companies and the NHIS, as disclosed by MOHW.

^c^ TOL, tolerance; VIF, variance inflation factor. TOL and VIF were calculated to assess multicollinearity among independent variables. A TOL value close to 1 and a VIF value below 10 indicate minimal multicollinearity.

^d^ t(p), t-statistic and *P*-value.

The full model identified the budget impact variable as the most significant predictor of the ‘KOR/A7 Lowest’ price ratio (β = −0.838, *P* < 0.001). This indicates that higher financial impact correlates with lower prices relative to the A7 minimum price. In the stepwise model, hybrid RSA contracts (Expenditure CAP + Refund) were positively associated with the price ratio (β = 0.595, *P* < 0.005), reflecting the role of RSA in balancing financial sustainability and access. The explanatory power of the model increased from 40.1% to 46.7%.

### 3.4 Hierarchical regression analysis for the “KOR/A7 lowest” price ratio

To verify the impact of the significant independent variables identified in the multiple regression analysis and the remaining variables on the explanatory power, a hierarchical regression analysis was performed. As summarized in Table 4, when the RSA variable (Model 2) was added to the model with the fixed budget impact variable (Model 1), the explanatory power increased from 0.215 to 0.499. However, when the remaining general variables were added (Model 3), the explanatory power did not increase further, and the model did not meet the assumptions of fit. The hierarchical regression analysis confirmed that RSA variables significantly improved the explanatory power (adjusted *R*-squared from 0.215 to 0.499). Adding general variables did not further enhance the model’s fit.

**TABLE 4:** Results of hierarchical regression analysis.

Variables		Model 1^c^			Model 2^c^			Model 3^c^		
		B	β	t(p)	B	β	t(p)	B	β	t(p)
Company	Domestic							Reference		
	Multi national							−0.018	−0.200	−1.083
Period	Before 2019							References		
	After 2019							0.003	0.046	0.164
ERP List^a^	A7							−0.001	−0.026	−0.121
	Rest							0.000	0.048	0.234
Indication	Non-Orphan							References		
	Orphan and Non-Cancer							0.007	0.104	0.302
	Orphan and Cancer							0.014	0.214	0.695
RSA	None				References					
	Cap				−0.021	−0.337	−1.648	−0.022	−0.345	−1.411
	Cap and Refund				0.023	0.355	1.683	0.020	0.312	0.827
Budget Impact^b^		−1.174 × 10^−6^	−0.492	−2.992^*^	−1.978 × 10^−6^	−0.829	−5.371^***^	−2.000 × 10^−6^	−0.838^***^	−4.634
Constant		0.973		146.8^***^	0.983		83.6^***^	0.988		26.7^***^
F statistic		8.950**			10.612^***^			3.158*		
Adjusted *R*-squared		0,215			0.499			0.401		
Durbin-Watson		2.540								

*P < 0.05, **P < 0.005, ***P < 0.001.

B, unstandardized regression coefficients; β, standardized regression coefficients; RSA, risk-sharing agreements; ERP, external reference price; A7, advanced countries (United States, United Kingdom, Italy, Germany, Japan, Switzerland, France).

^a^ ERP List: The number of listed countries, classified as A7 and OECD countries.

^b^ Budget Impact: Values are described as million KRW (Sum, Mean, Standard Deviation) or rank statistics (Mean Rank, Ranks Sum). Budget Impact represents the estimated annual sales reported during pricing negotiations between pharmaceutical companies and the NHIS, as disclosed by MOHW.

^c^ TOL, tolerance; VIF, variance inflation factor. TOL and VIF were calculated to assess multicollinearity among independent variables. A TOL value close to 1 and a VIF value below 10 indicate minimal multicollinearity.

## 4 Discussion

This study identified that the introduction of high-cost drugs under the PEE system has led to significant financial challenges, despite improving patient access. Our analysis revealed that the budget impact variable strongly influenced drug pricing, with higher financial impact correlating with lower price ratios compared to the A7 minimum price. These findings suggest a delicate balance between affordability and accessibility that requires refined pricing and negotiation strategies.

The analysis confirmed that drug prices in Korea are closely aligned with the A7 minimum price, with no significant reductions achieved through the PEE system. This underscores the importance of adopting flexible pricing models and innovative negotiation strategies to mitigate financial uncertainties while maintaining equitable patient access.

The financial impact of PEE drugs has grown significantly, accounting for a substantial increase in pharmaceutical expenditures from 2015 to 2022. This trend highlights the need for institutional reforms to manage such growth effectively. Allocating a dedicated portion of the pharmaceutical budget to PEE drugs and implementing tailored RSA contracts could be potential solutions.

The regression analysis demonstrated that higher financial impact is associated with lower price ratios compared to the A7 minimum price, indicating strategic pricing efforts to balance affordability and sustainability. Further monitoring is required to assess trends beyond 2022 and to refine strategies for managing high-cost drugs effectively.

The ERP ratio variables related to price ratios showed a strong positive correlation, suggesting that global pricing trends are interconnected during the global introduction of new drugs. As a result, the Korean government is working to set new PEE drug prices below the A7 benchmark. However, the sharp rise in the total financial impact and the inclusion of non-rare drugs since 2019 signal a critical turning point in the management of total pharmaceutical expenditure, requiring significant adjustments.

Despite the insights provided by this study, several limitations must be acknowledged. First, identifying the most appropriate method for establishing causal relationships among variables through regression analysis is challenging. Although multiple variables were significant in the univariate analysis, causal relationships were not confirmed, potentially due to the small number of new PEE drugs and the inherent uncertainty in drug pricing. Previous studies have highlighted variations among government personnel in transparency during drug price negotiations (Kwon and Kim, 2020). Furthermore, while we referred to HIRA and NHIS guidelines to verify ERP variables, matching prices from other OECD countries with A7 drug prices was difficult. HIRA’s reliance on A7 average and lowest prices complicates direct comparisons with NHIS data, which often references broader international pricing benchmarks. This highlights the need for regular updates and alignment of ERP methodologies across institutions to improve pricing consistency. Regular updates of drug price information from countries outside the A7 by NHIS are crucial for the accurate verification of ERP variables. Lastly, the Budget Impact variable, derived from annual expected sales reported in official MOHW data, serves as a proxy for the financial burden of new PEE drugs on the healthcare system and reflects their anticipated resource allocation needs. It should not be conflated with actual health insurance expenditures. These limitations highlight the necessity for future research to align ERP methodologies and collect real-world expenditure data to refine pricing models under the PEE system.

This study’s focus on the relationship between price and financial impact during the inclusion of new PEE drugs makes annual expected sales a more appropriate variable. Future research should aim to collect real-world health insurance expenditure data on new PEE drugs to reassess the PEE system and establish more rational pricing negotiations and financial management systems.

The PEE system has notably improved patient access to high-cost drugs. However, the rising expenditure on pharmaceuticals has made budget impact a significant consideration in pricing decisions, highlighting the need for ongoing monitoring of drug expenditure by payers. As the system evolves, enhanced oversight and policy adjustments will be crucial for balancing cost containment with equitable patient access. In this context, developing tiered RSA models based on drug classification or therapeutic impact could be a viable approach to achieving this balance.

## Data Availability

The raw data supporting the conclusions of this article will be made available by the authors, without undue reservation.
